# Analysis of Nanofluid Particles in a Duct with Thermal Radiation by Using an Efficient Metaheuristic-Driven Approach

**DOI:** 10.3390/nano12040637

**Published:** 2022-02-14

**Authors:** Naveed Ahmad Khan, Muhammad Sulaiman, Carlos Andrés Tavera Romero, Fahad Sameer Alshammari

**Affiliations:** 1 Department of Mathematics, Abdul Wali Khan University, Mardan 23200, Pakistan; ahmednaveed854477@gmail.com; 2 COMBA I + D Research Group of Universidad Santiago de Cali, Santiago de Cali 760036, Colombia; carlos.tavera00@usc.edu.co; 3 Department of Mathematics, College of Science and Humanities in Alkharj, Prince Sattam bin Abdulaziz University, Al-Kharj 11942, Saudi Arabia; f.alshammari@psau.edu.sa

**Keywords:** porous semipermeable duct, steady two-phase flow, nanofluid, Buongiorno model, magnetic field, artificial intelligence, arithmetic optimization algorithm, soft computing

## Abstract

This study investigated the steady two-phase flow of a nanofluid in a permeable duct with thermal radiation, a magnetic field, and external forces. The basic continuity and momentum equations were considered along with the Buongiorno model to formulate the governing mathematical model of the problem. Furthermore, the intelligent computational strength of artificial neural networks (ANNs) was utilized to construct the approximate solution for the problem. The unsupervised objective functions of the governing equations in terms of mean square error were optimized by hybridizing the global search ability of an arithmetic optimization algorithm (AOA) with the local search capability of an interior point algorithm (IPA). The proposed ANN-AOA-IPA technique was implemented to study the effect of variations in the thermophoretic parameter (Nt), Hartmann number (Ha), Brownian (Nb) and radiation (Rd) motion parameters, Eckert number (Ec), Reynolds number (Re) and Schmidt number (Sc) on the velocity profile, thermal profile, Nusselt number and skin friction coefficient of the nanofluid. The results obtained by the designed metaheuristic algorithm were compared with the numerical solutions obtained by the Runge–Kutta method of order 4 (RK-4) and machine learning algorithms based on a nonlinear autoregressive network with exogenous inputs (NARX) and backpropagated Levenberg–Marquardt algorithm. The mean percentage errors in approximate solutions obtained by ANN-AOA-IPA are around $10^{-6}$ to $10^{-7}$. The graphical analysis illustrates that the velocity, temperature, and concentration profiles of the nanofluid increase with an increase in the suction parameter, Eckert number and Schmidt number, respectively. Solutions and the results of performance indicators such as mean absolute deviation, Theil’s inequality coefficient and error in Nash–Sutcliffe efficiency further validate the proposed algorithm’s utility and efficiency.

## 1. Introduction

Nanofluids are defined as fluids that contain nanometer-sized particles (less than 100 nanometers (nm) in size), which are suspended in base fluids to enhance their convectional heat transfer. Nanofluidic problems of the flow and heat transfer characteristics are important from a theoretical as well as a practical point of view, and they have been extensively studied in applied sciences and various engineering applications, such as thermal power generation systems, the cooling of a large metallic plate in a bath, fiber spinning, glass blowing, melt spinning, wire coating dynamics and the extrusion of material through a die [1]. Choi and Eastman [2] were among the first to introduce nanoparticles to the fluid system. The basic idea was based on the ability of nanoparticles to improve heat transfer in classical base fluids, which suggested the potential to use nanofluids in advanced thermal systems with low economical cost. Some well-known examples of nanoparticles include aluminum (Al), copper (Cu), ferric oxide $\left({\mathrm{Fe}}_{2}O_{3}\right)$, alumina $\left({\mathrm{Al}}_{2}O_{3}\right)$, silicon dioxide $\left({\mathrm{SiO}}_{2}\right)$ and carbon nanotubes (CNTs). The suspension of base fluids such as water, oil and ethylene glycol in various nanofluids is an effective way to achieve a high heat transfer rate in fluid systems [3,4]. Sheikholeslami [5] analyzed the various shapes of aluminum oxide $\left({\mathrm{Al}}_{2}O_{3}\right)$ using the Darcy porous medium with thermal radiation. Further, he investigated nanoparticles with different shapes, such as copper oxide and water with Brownian motion, revealing that platelet-shaped nanoparticles had an immense impact when compared with other nanoparticle shapes [6].

In recent times, nanofluids have gained researchers’ attention due to their vast applications and their impact when combined with base fluids. In 2014, Rashidi [7] investigated the incompressible electrically conductive nanofluid flow over a porous rotating disk by applying the second law of thermodynamics. It was concluded that magnetic spinning disk drives have important applications in improving heat transfer in renewable energy systems. Vajravelua and Kumar [8] studied the numerical solutions and impact of physical parameters on the viscous flow of a magnetohydrodynamic (MHD) nanofluid in a rotating system with porous and stretched plates. The thermal performance and heat sink of a rectangular microchannel containing nanoparticles such as copper, zinc and aluminum oxide in ethylene glycol fluid were numerically investigated by Seyf and Nikaaein [9]. In addition, a nanofluid was used by Jang and Choi [10] to study cooling performance. Their results demonstrated that the nanofluid reduced the thermal resistance and difference in temperatures in a heated wall of microchannels. Rout [11] analyzed kerosene oil-based and water-based copper between two parallel plates with thermal radiation. Nanofluid flow in horizontal spiral coils used for solar ponds was studied by Khodabandeh [12,13]. Ahmed [14] numerically studied the unsteady radiative flow of a chemically reacting fluid over a convectively heated stretchable surface with cross-diffusion gradients.

Generally, mathematical models of problems involving nanofluid flow are highly nonlinear in nature. Therefore, various methods in the literature have been developed to tackle such problems. The optimal homotopy analysis method (OHAM) has been used to study numerical solutions of the Cattaneo–Christov heat flux model [15,16], nanofluids over a nonlinear stretching surface with variable surface thickness [17], the flow and heat transfer of nanofluids over a moving surface with nonlinear velocity [18] and non-Newtonian nanofluid flow in porous media with gyrotactic microorganisms [19,20]. M. Govindaraju [21] investigated the boundary layer flow of gold–thorium water based on nanofluids over a moving semi-infinite plate by using the homotopy perturbation method (HPM). YAS El-Masry [22] studied the impacts of varying magnetic field and free convection heat transfer on Eyring–Powell nanofluid flow with peristalsis by using the variational iteration method (VIM). Thumma [23] used the Adomian decomposition method (ADM) for a Cu/CuO–water viscoplastic nanofluid over a porous stretched sheet. All of these techniques are based on traditional deterministic approaches that have their own advantages and limitations in terms of solution quality, convergence rate and applicability domain. However, stochastic metaheuristic techniques developed through artificial intelligence algorithms have not been explored and exploited for solving nonlinear models of nanofluids. Recently, the strength of stochastic techniques based on artificial neural networks (ANNs) using bio- and nature-inspired computing paradigms has been extensively applied to study the approximate solutions of stiff nonlinear problems, such as the saturation of oil and water during the secondary oil recovery process [24], the bath of a wire during coating with Oldroyd 8-constant fluid [25], the rolling motion of ships in random beam seas [26], the study of 3-D Prandtl nanofluid flow over a convectively heated sheet [27], nonlinear problems arising in heat transfer [28,29], thermal radiation and Hall effects on the boundary layer flow of a nanofluid [30] and the Lorenz chaotic attractor (LCA) and double-scroll attractor (DSA) in secure communication systems [31]. Some salient features of the designed schemes are as follows:

- In this study, a mathematical model of the steady two-phase flow of a nanofluid in a semipermeable duct in the presence of external forces was formulated by using the Buongiorno model and basic concepts of continuity and momentum equations. Further, the model was reduced to a system of ordinary differential equations.
- Moreover, to study the effect of variations in certain parameters, such as the thermophoretic parameter (Nt), Hartmann number (Ha), Brownian (Nb) and radiation (Rd) motion parameters, Eckert number (Ec), Reynolds number (Re) and Schmidt number (Sc), on the velocity profile, thermal profile, Nusselt number and skin friction coefficient of the nanofluid, a soft computing metaheuristic technique was designed. The intelligent computational strength of artificial neural networks was utilized with a combination of unsupervised and supervised learning strategies.
- The results obtained with the proposed ANN-AOA-IPA technique were compared with the methods available in the latest literature. In addition, to study the convergence and stability of the results, the proposed algorithm was implemented for 100 independent runs.
- Extensive graphical, statistical and sensitivity analyses were conducted to study the errors in approximate solutions based on mean absolute deviations, Theil’s inequality coefficient and error in Nash–Sutcliffe efficiency.

The rest of the paper is organized as follows: In Section 2, we analyze the mathematical model of a nanofluid migrating in a semipermeable duct, and then we describe the proposed methodology in Section 3 for solving the governing equations of the problem. In Section 4, we discuss the numerical simulation and results obtained by executing the proposed technique for 100 runs. Finally, we conclude the paper in Section 5.

## 2. Mathematical Formulation

**Figure 1:**
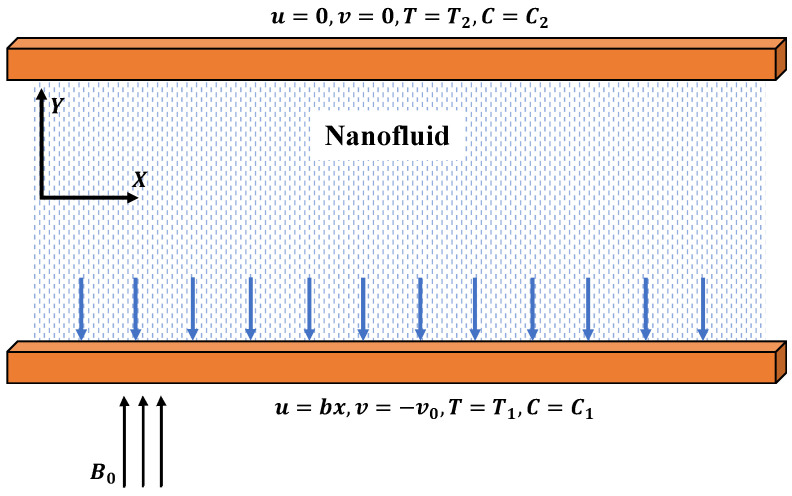
Steady two-phase flow of the nanofluid.

Consider the steady two-phase flow of a nanofluid in a semipermeable duct, as shown in Figure 1. It is assumed that the upper surface is cold, while the lower surface is hot. In addition, the effects of Joule heating and radiation on temperature distribution along with the constant vertical magnetic field $B_{0}$ are applied. The basic governing equations are as follows [32,33]:

(1) $$\frac{\partial v}{\partial y}+\frac{\partial u}{\partial x}=0,$$

(2) $$\rho_{f}\left(v\frac{\partial u}{\partial y}+u\frac{\partial u}{\partial x}\right)-\mu \left(\frac{\partial^{2}u}{\partial y^{2}}\right)+\frac{\partial p}{\partial x}+\sigma B_{0}^{2}u=0,$$

(3) $$\left(u\frac{\partial T}{\partial x}+v\frac{\partial T}{\partial y}\right){\left(\rho C_{p}\right)}_{f}+\frac{\partial q_{r}}{\partial y}-\sigma B_{0}^{2}u^{2}=k\left(\frac{\partial^{2}T}{\partial y^{2}}\right)+{\left(\rho C_{p}\right)}_{p}\left[D_{B}\left\{\frac{dC}{\;dy}\cdot \frac{dT}{\;dy}\right\}+\left(D_{T}/T_{2}\right)\left\{{\left(\frac{dT}{\;dy}\right)}^{2}\right\}\right],$$

(4) $$q_{r}=-\frac{4\sigma_{e}}{3\beta_{R}}\frac{\partial T^{4}}{\partial y},$$

(5) $$\frac{\partial C}{\partial y}v+\frac{\partial C}{\partial x}u=\left(\frac{D_{T}}{T_{2}}\right)\left\{\frac{d^{2}C}{\;dy^{2}}\right\}+D_{B}\frac{\partial^{2}C}{\partial y^{2}}.$$

According to Raptis [34], the fluid temperature is

(6) $$T^{4}≅4T_{c}^{3}T-3T_{c}^{4},$$

subject to boundary conditions

(7) $$C=C_{1},u=bx,v=-v_{0},T=T_{1}\;\mathrm{at}\;y=-a,$$

(8) $$C=C_{2},v=0,u=0,T=T_{2}\;\mathrm{at}\;y=a,$$

where $C_{p}$ represents the specific heat capacity, $B_{0}$ is the magnetic field, μ is dynamic viscosity, σ is electrical conductivity, *u* and *v* are horizontal and vertical velocities, $q_{r}$ is thermal radiation, $\rho_{e}$ is the Stefan–Boltzmann constant, *T* is thermal quantity, and $\beta_{R}$ is the mean absorption coefficient. The following parameters are used to convert the above equations into ordinary differential equations [32].

(9) $$\eta =\frac{y}{a},\;u=bx\frac{df}{d\eta },\;v=-abf\left(\eta \right),\;\theta =\frac{T-T_{1}}{T_{1}-T_{2}},\;\phi =\frac{C-C_{1}}{C_{1}-C_{2}}.$$

Using Equation (9) will result in a system of ordinary differential equations, which are given as

(10) $$\frac{df^{4}}{d\eta^{4}}+Re\left(f\frac{df^{3}}{d\eta^{3}}-\frac{df^{2}}{d\eta^{2}}\frac{df}{d\eta }\right)-Ha^{2}\frac{df^{2}}{d\eta^{2}}=0,$$

(11) $$\left(1+\frac{4}{3}Rd\right)\frac{d\theta^{2}}{d\eta^{2}}+Prf\frac{d\theta }{d\eta }+Ha^{2}Ec\frac{Pr}{Re}{\left(\frac{df}{d\eta }\right)}^{2}+Nb\frac{d\theta }{d\eta }\frac{d\phi }{d\eta }+Nt{\left(\frac{d\theta }{d\eta }\right)}^{2}=0,$$

(12) $$\frac{d\phi^{2}}{d\eta^{2}}+Sc\left(\frac{d\phi }{d\eta }\right)+\frac{Nt}{Nb}\frac{d\theta^{2}}{d\eta^{2}}=0,$$

where Re, Ha, Rd, Ec, Pr, Nt, Nb and Sc are the Reynolds number, Hartmann number, radiation motion parameter, Brownian motion parameter, Eckert number, Prandtl number, thermophoretic parameter and Schmidt number, respectively, which are defined as

(13) $$Re=\frac{a^{2}b}{v},\;Ha=B_{0}a\sqrt{\frac{\sigma }{\mu }},\;Rd=4\sigma_{e}T_{c}^{3}/\left(\beta_{R}K\right),\;Pr={\left(\rho C_{p}\right)}_{f}\frac{a^{2}b}{k},\;Sc=\frac{v}{D},$$

(14) $$Nb=\Delta C\alpha^{-1}D_{B}{\left(\rho C_{p}\right)}_{f}^{-1}{\left(\rho C_{p}\right)}_{p},\;Nt=\frac{\Delta T{\left(\rho C_{p}\right)}_{p}D_{T}}{{\left(\rho C_{p}\right)}_{f}\alpha },\;Ec=\frac{\rho_{f}{\left(bx\right)}^{2}}{\left(\rho C_{p}\right)\Delta T}.$$

The corresponding boundary conditions for Equations (10)–(12) are

(15) $$\begin{array}{cc} f(1) & =0,f(-1)=\lambda ,\\ f^{\prime }\left(1\right) & =0,f^{\prime }\left(-1\right)=1,\\ \theta (1) & =0,\theta (-1)=1,\\ \phi (-1) & =1,\phi (1)=0. \end{array}$$

The Nusselt number (Nu) and specific heat over the bottom wall are defined as

(16) $$Nu=\left|\theta^{\prime }\left(-1\right)\right|,C_{f}=\left|f^{\prime \prime }\left(-1\right)\right|.$$

## 3. Methodology

In this section, we discuss the designed methodology for the solutions of the migration of nanoparticles in a duct with variable thermal radiation. The proposed technique consists of two phases. Initially, unsupervised ANN models in terms of mean square error (MSE) are constructed with the log-sigmoid activation function for Equations (10)–(12). In the second phase, the parameters involved in the ANN model are optimized for the solutions of governing equations by using global search and local search techniques.

### 3.1. Neural Network Modeling

ANN-based models have been extensively used to study the approximate solutions of various problems arising in engineering and applied sciences [24,33,35]. The mathematical model for solutions of the steady two-phase flow of a nanofluid in the duct is given by a feed-forward ANN in the form of continuous mapping, which is defined as

(17) $$f\left(\eta \right)=\sum_{i=1}^{k}\alpha_{i}h\left(\omega_{i}\eta +\beta_{i}\right),$$

(18) $$\theta \left(\eta \right)=\sum_{i=1}^{k}\alpha_{i}h\left(\omega_{i}\eta +\beta_{i}\right),$$

(19) $$\phi \left(\eta \right)=\sum_{i=1}^{k}\alpha_{i}h\left(\omega_{i}\eta +\beta_{i}\right).$$

In general, the nth-order derivative of the above models in terms of input, hidden and output layers is given as

(20) $$f^{\left(n\right)}\left(x\right)=\sum_{i=1}^{k}\alpha_{i}h^{\left(n\right)}\left(\omega_{i}\eta +\beta_{i}\right).$$

In Equations (17)–(20), log-sigmoid is used as an activation function in the hidden layer; then, the updated solutions and their nth order can be written as

(21) $$f\left(\eta \right)=\sum_{i=1}^{k}\alpha_{i}\left(\frac{1}{1+e^{-\left(\omega_{i}\eta +\beta_{i}\right)}}\right),$$

(22) $$f^{\left(n\right)}\left(\eta \right)=\sum_{i=1}^{k}\alpha_{i}{\left(\frac{1}{1+e^{-\left(\omega_{i}\eta +\beta_{i}\right)}}\right)}^{\left(n\right)},$$

where $\alpha =\left[\alpha_{1},\alpha_{2},\alpha_{3},\ldots ,\alpha_{k}\right],\omega =\left[\omega_{1},\omega_{2},\omega_{3},\ldots ,\omega_{k}\right]$ and $\beta =\left[\beta_{1},\beta_{2},\beta_{3},\ldots ,\beta_{k}\right]$ are the optimization decision weights that are to be found during the course of calculating the solution using the arithmetic optimization algorithm and interior point algorithm.

Further, the suggested closed-form solutions and their derivatives are used to construct the fitness functions in terms of mean square error for the governing model of the problem along with the boundary conditions:

(23) $$\;\mathrm{minimize}\;\Theta =\Theta_{1}+\Theta_{2}+\Theta_{3}+\Theta_{4},$$

where $\Theta_{1},\Theta_{2}$ and $\Theta_{3}$ correspond to differential equations, and $\Theta_{4}$ represents the boundary conditions, which are defined as

(24) $$\Theta_{1}=\frac{1}{M}\sum_{i=1}^{M}{\left(\frac{df_{i}^{4}}{d\eta^{4}}+Re\left(f_{i}\frac{df_{i}^{3}}{d\eta^{3}}-\frac{df_{i}^{2}}{d\eta^{2}}\frac{df_{i}}{d\eta }\right)-Ha^{2}\frac{df_{i}^{2}}{d\eta^{2}}\right)}^{2},$$

(25) $$\Theta_{2}=\frac{1}{M}\sum_{i=1}^{M}{\left(\left(1+\frac{4}{3}Rd\right)\frac{d\theta_{i}^{2}}{d\eta^{2}}+\mathrm{Pr}f_{i}\frac{d\theta_{i}}{d\eta }+Ha^{2}Ec\frac{Pr}{Re}{\left(\frac{df_{i}}{d\eta }\right)}^{2}+Nb\frac{d\theta_{i}}{d\eta }\frac{d\phi_{i}}{d\eta }+Nt{\left(\frac{d\theta_{i}}{d\eta }\right)}^{2}\right)}^{2},$$

(26) $$\Theta_{3}=\frac{1}{M}\sum_{i=1}^{M}{\left(\frac{d\phi_{i}^{2}}{d\eta^{2}}+Sc\left(\frac{d\phi_{i}}{d\eta }\right)+\frac{Nt}{Nb}\frac{d\theta_{i}^{2}}{d\eta^{2}}\right)}^{2},$$

(27) $$\Theta_{4}=\frac{1}{8}\left(\begin{array}{c} {\left(f\left(1\right)\right)}^{2}+{\left(f\left(-1\right)-\lambda \right)}^{2}+{\left(f^{\prime }\left(1\right)\right)}^{2}+{\left(f^{\prime }\left(-1\right)-1\right)}^{2}\\ +{\left(\theta \left(1\right)\right)}^{2}+{\left(\theta \left(-1\right)-1\right)}^{2}+{\left(\phi \left(1\right)\right)}^{2}+{\left(\phi \left(-1\right)-1\right)}^{2} \end{array}\right),$$

where M=1/h. The unsupervised fitness function given in Equation (23) is optimized by an optimization algorithm to find the values of neurons in the ANN structure.

### 3.2. Optimization Methods

#### 3.2.1. Arithmetic Optimization Algorithm

**Figure 2:**
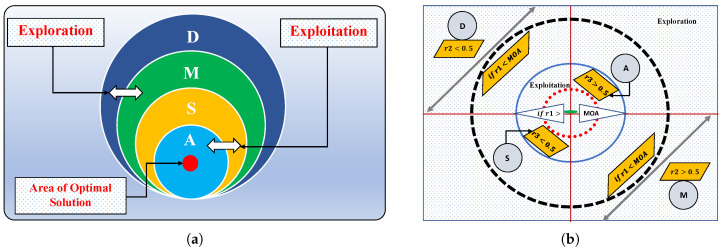
(**a**) Hierarchy of arithmetic operators (dominance decreases from the top down) and (**b**) the search phases of the AOA.

The arithmetic optimization algorithm (AOA) is a metaheuristic technique proposed by Abualigah [36] in 2021 and was inspired by basic arithmetic operators in mathematics, i.e., multiplication (M,×), division (D,÷), subtraction (S,−) and addition (A,+). Figure 2 shows the dominance of the operators from outside to inside, along with an overview of the search mechanism of AOA. This algorithm is a population-based technique that is used to find the optimal solutions of a problem without calculating the gradient. The parameter settings for the execution of AOA are given in Table 1.

The arithmetic optimization algorithm begins the process of optimization by randomly generating a set of N candidate solutions *X*, which is given as

(28) $$X=\left[\begin{array}{cccccc} x_{1,1} & \cdots & \cdots & x_{1,j} & x_{1,n-1} & x_{1,n}\\ x_{2,1} & \cdots & \cdots & x_{2,j} & \cdots & x_{2,n}\\ \cdots & \cdots & \cdots & \cdots & \cdots & \cdots \\ \vdots & \vdots & \vdots & \vdots & \vdots & \vdots \\ x_{N-1,1} & \cdots & \cdots & x_{N-1,j} & \cdots & x_{N-1,n}\\ x_{N,1} & \cdots & \cdots & x_{N,j} & x_{N,n-1} & x_{N,n} \end{array}\right].$$

In the next step, AOA starts to improve the candidate solution by choosing the search mechanism (i.e., exploration or exploitation), so the math optimizer accelerated (*MOA*) function is used to determine the coefficient, which is defined as

(29) $$MOA\left(t\right)=\mathrm{Min}+t\times \left(\frac{\mathrm{Max}-\mathrm{Min}}{T}\right)$$

where *T* represents the total number of iterations, MOA(t) is the function value at the *t*th iteration, and t∈[1,T] is the current iteration. Max and Min denote the maximum and minimum values of the accelerated function, respectively.

**Figure 3:**
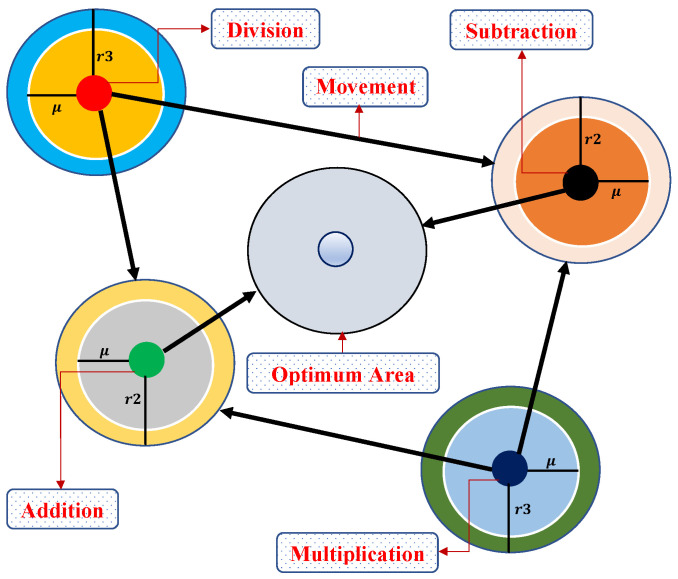
Model of updating the position of math operators in AOA toward the optimum area.

Exploration phase:

In this phase, the exploratory behavior of AOA is established. In mathematical calculations, arithmetic operators such as multiplication and division have high distribution values or decisions (relative to other operators) that are committed to the exploration search mechanism. Therefore, division or multiplication search strategies are used by AOA to explore the candidate space and find a better solution. This phase of searching is shown in Figure 3. The phenomenon is modeled as

(30) $$x_{i,j}\left(t+1\right)=\left\{\begin{array}{cc} \mathrm{best}\left(x_{j}\right)÷\left(MOP+\epsilon \right)\times \left(\left(UB_{j}-LB_{j}\right)\times \bar{\mu }+LB_{j}\right), & r2<0.5\\ \mathrm{best}\left(x_{j}\right)\times MOP\times \left(\left(UB_{j}-LB_{j}\right)\times \bar{\mu }+LB_{j}\right) & \;\mathrm{otherwise}\; \end{array}\right.$$

This search is performed if r1>MOA, where *r*1 is a random number. Then, D will be executed if r2<0.5; otherwise, M will be incorporated. Here, $x_{i}\left(t+1\right)$ is the *i*th solution, $x_{i,j}\left(t\right)$ denotes the *j*th position of the *i*th solution at current iteration, $UB_{j}$ and $LB_{j}$ are the upper and lower bounds of the *j*th position, and $\bar{\mu }$ is a controlling parameter equal to 0.5 and is used to tune the exploration search phase. In addition, a coefficient known as math optimizer probability (*MOP*) is defined, in which the sensitive parameter $\bar{\alpha }=5$ is used for the accuracy of the iteration in this phase.

(31) $$MOP\left(t\right)=1-\frac{t^{\left(\frac{1}{\bar{\alpha }}\right)}}{T^{\left(\frac{1}{\bar{\alpha }}\right)}}$$

Exploitation phase:

In this phase, the search space is exploited in depth to find the optimum solutions around the candidate space. If r1≤MOA, exploitation is activated, in which subtraction and addition from arithmetic are utilized in the mathematical model for updating the positions of the solution candidates, which is given as

(32) $$x_{i,j}\left(t+1\right)=\left\{\begin{array}{cc} \mathrm{best}\left(x_{j}\right)-MOP\times \left(\left(UB_{j}-LB_{j}\right)\times \bar{\mu }+LB_{j}\right), & r3<0.5\\ \mathrm{best}\left(x_{j}\right)+MOP\times \left(\left(UB_{j}-LB_{j}\right)\times \bar{\mu }+LB_{j}\right) & \;\mathrm{otherwise}\; \end{array}\right.$$

Figure 3 explains how a search solution updates its positions according to arithmetic operators in the 2-dimensional search space. It can be seen that D, M, S and A estimate the position of the near-optimal solution, and other solutions update their positions stochastically around the area of the near-optimal solution [37]. Some recent applications of AOA include workflow scheduling [38], cooling, heating and power systems [39], the forced switching mechanism [38] and the identification of proton exchange membrane fuel cells [40].

#### 3.2.2. The Proposed Hybridized Algorithm

**Figure 4:**
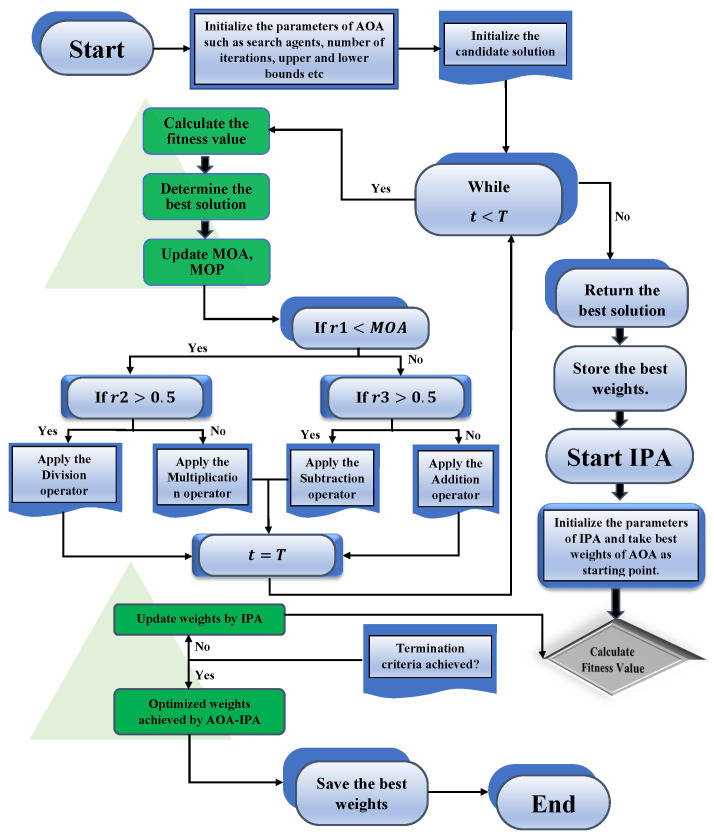
An overview of the mechanism of AOA and IPA for finding the solution of the system of differential equations representing the moment of nanoparticles in a permeable duct.

Metaheuristic (MH) algorithms are high-level unsupervised learning techniques that are developed to solve complex optimization problems arising in various fields of physics, engineering, mathematics and medical sciences. MH algorithms are flexible, concise and vital in calculating solutions. However, in addition to these merits, they have some drawbacks. For instance, when dealing with complex optimization problems, AOA only utilizes the information of the best position in the population, which sometimes causes it to become trapped in a local optimum, which might affect the speed of the convergence of the algorithm. Therefore, the aim of this study was to develop a new hybridized algorithm to enhance the speed of the convergence of solutions in the local search phase. The solutions obtained by AOA are further tuned by a local search technique known as the interior point algorithm (IPA). It is used for the optimization of constrained and unconstrained optimization problems. Some recent applications of IPA include determining the solution of nonsymmetric exponential-cone optimization [41], convex quadratic programming [42], simulation of viscoplastic fluid flows [43] and simulation of aircraft parts riveting [44]. The detailed workflow of the proposed ANN-AOA-IPA is shown in Figure 4.

## 4. Numerical Experimentation and Discussion

The proposed ANN-AOA-IPA algorithm was implemented to study the effect of variations in various parameters, such as the thermophoretic parameter (Nt), Hartmann number (Ha), Brownian (Nb) and radiation (Rd) motion parameters, Eckert number (Ec), Reynolds number (Re) and Schmidt number (Sc), on the velocity profile, thermal profile, Nusselt number and skin friction coefficient of the nanofluid. Each parameter is varied while keeping the other parameters fixed at values of Ha=1.0,λ=1.0,Rd=0.5, Ec=0.5,Sc=1.0,Nt=0.001,Nb=0.01 and Pr=10.

The approximate solutions obtained by the designed scheme are compared with the numerical solutions obtained by RK-4 and results obtained by machine learning algorithms, as shown in Table 2. These solutions can be regenerated by using the closed-form solution given in Appendix A. In addition, Table 3 shows the accuracy and stability of the solutions in terms of absolute errors. It can be seen that the approximate solution overlaps with the analytical solution, with minimum absolute errors between $10^{-4}$ to $10^{-9}$ and $10^{-5}$ to $10^{-8}$, respectively.

**Figure 5:**
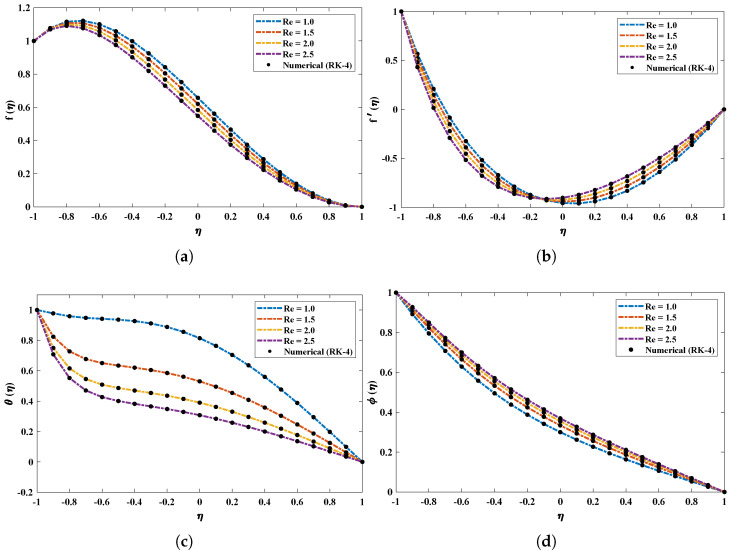
(**a**–**d**) The influence of variations in Reynolds number on velocity, temperature and concentration profiles of the nanofluid subjected to the magnetic field.

**Figure 6:**
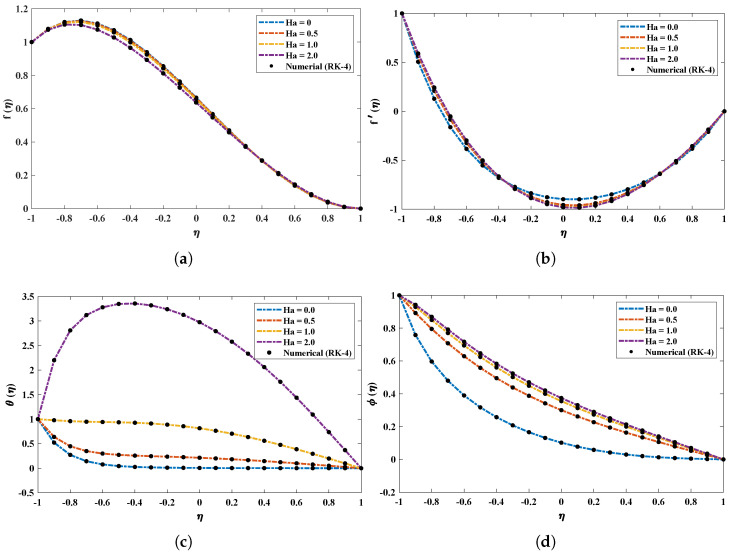
(**a**–**d**) Analysis based on the influence of variations in Hartman number on different profiles of the nanofluid.

Figure 5 demonstrates the effect of variations in the Reynolds number (Re) on profiles of *f*, $f^{\prime }$, θ and ϕ. It is observed that the vertical velocity (f) and temperature profiles (θ) possess an inverse relation with Re. Near the bottom wall, the horizontal velocity decreases, while it increases at the upper wall. In addition, ϕ increases with an increase in Re. The influence of the Hartman number (Ha) on the model is shown in Figure 6. Significant increases in the temperature and concentration profiles of the nanofluid are observed with an increase in Ha, while the velocity profile slightly decreases.

**Figure 7:**
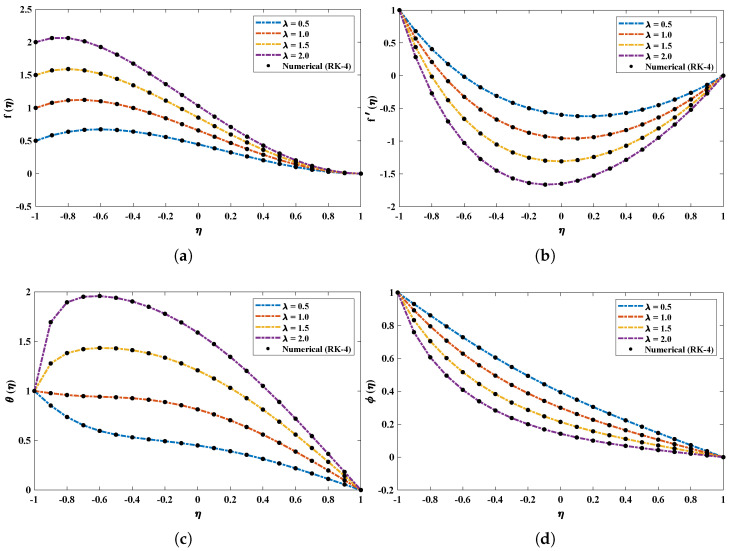
(**a**–**d**) The behavior of velocity, temperature and concentration of the nanoparticles when the suction parameter is varied from 0.5 to 2.

**Figure 8:**
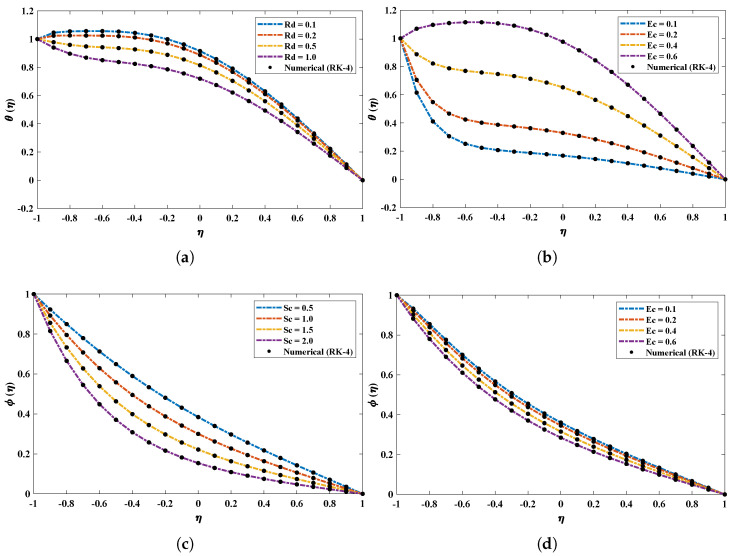
(**a**–**d**) The influence of variations in radiation parameter, Eckert number and Schmidt number on temperature and concentration profiles of the fluid.

**Figure 9:**
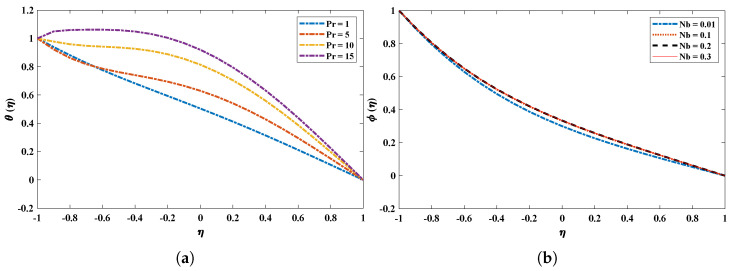
The effect of variations in (**a**) Prandtl number and (**b**) Brownian motion parameter on temperature and concentration profiles of the nanofluid.

The suction parameter λ was varied from 0.5 to 2.0 to study its effect on the velocity, thermal and concentration profiles of the nanofluid. The vertical velocity and temperature profiles increase with an increase in λ. From Figure 7 it is observed that the minimum velocity point shifts to the lower wall. In addition, the concentration of the fluid decreases. Figure 8 and Figure 9 illustrate the influence of variations in the radiation parameter, Eckert number, Schmidt number, Brownian motion parameter and Prandtl number on temperature and concentration profiles. It is concluded that increases in Rd, Ec and Sc cause decreases in θ and ϕ. In addition, an increase in the Brownian motion parameter increases the temperature profile of the nanofluid. It is also observed that the results obtained by the proposed technique overlap with the analytical solution.

**Figure 10:**
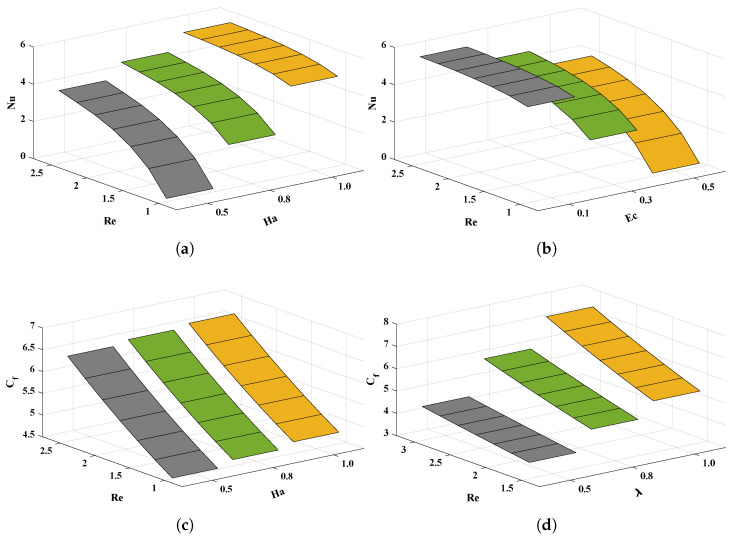
(**a**–**d**) The influence of variations in Reynolds number, Hartmann number, Eckert number and suction parameter on Nusselt number and skin friction coefficient.

Furthermore, graphical analyses in Figure 10 show the influence of variations in various parameters on the Nusselt number (Nu) and skin friction coefficient $C_{f}$. The values of the Nusselt number (Nu) and skin friction coefficient increase with increases in the Hartman number and suction parameter. The Nusselt number decreases with an increase in the Eckert number.

To check the performance of the proposed algorithm in terms of stability, consistency and accuracy, ANN-AOA-IPA was implemented for 100 independent runs. Various performance indicators were defined to check the validity of the solutions. The formulation of these parameters (mean absolute deviation, Theil’s inequality coefficient (TIC) and error in Nash–Sutcliffe efficiency (ENSE)) are given as

(33) $$\left[MAD_{f},MAD_{\theta },MAD_{\phi }\right]={\left[\begin{array}{c} \frac{1}{M}\sum_{j=1}^{M}\left|\bar{f}\left(\eta_{j}\right)-f\left(\eta_{j}\right)\right|,\\ \frac{1}{M}\sum_{j=1}^{M}\left|\bar{\theta }\left(\eta_{j}\right)-\theta )\left(\eta_{j}\right)\right|,\\ \frac{1}{M}\sum_{i=1}^{M}\left|\bar{\phi }\left(\eta_{j}\right)-\phi \left(\eta_{j}\right)\right|, \end{array}\right]}^{t}$$

(34) $${\left[\;\mathrm{TIC}{\;}_{f},\;\mathrm{TIC}{\;}_{\theta },\;\mathrm{TIC}{\;}_{\phi }\right]=[\begin{array}{c} \frac{\sqrt{\frac{1}{M}\sum_{j=1}^{M}{\left(\bar{f}\left(\eta_{j}\right)-f\left(\eta_{j}\right)\right)}^{2}}}{\sqrt{\frac{1}{M}\sum_{j=1}^{M}{\left(\bar{f}\left(\eta_{j}\right)\right)}^{2}}+\sqrt{\frac{1}{M}\sum_{j=1}^{M}{\left(f\left(\eta_{j}\right)\right)}^{2}}},\\ \frac{\sqrt{\frac{1}{M}\sum_{j=1}^{M}{\left(\bar{\theta }\left(\eta_{j}\right)-\theta \left(\eta_{j}\right)\right)}^{2}}}{\sqrt{\frac{1}{M}\sum_{j=1}^{M}{\left(\bar{\theta }\left(\eta_{j}\right)\right)}^{2}}+\sqrt{\frac{1}{M}\sum_{j=1}^{M}{\left(\theta \left(\eta_{j}\right)\right)}^{2}}},\\ \frac{\sqrt{\frac{1}{M}\sum_{j=1}^{M}{\left(\bar{\phi }\left(\eta_{j}\right)-\phi \left(\eta_{j}\right)\right)}^{2}}}{\sqrt{\frac{1}{M}\sum_{j=1}^{M}{\left(\bar{\phi }\left(\eta_{j}\right)\right)}^{2}}+\sqrt{\frac{1}{M}\sum_{j=1}^{M}{\left(\phi \left(\eta_{j}\right)\right)}^{2}}}, \end{array}]}^{t},$$

(35) $$\left[NSE_{f},NSE_{\theta },NSE_{\phi }\right]={\left[\begin{array}{cc} 1-\frac{\frac{1}{M}\sum_{j=1}^{M}{\left(\bar{f}\left(\eta_{j}\right)-f\left(\eta_{j}\right)\right)}^{2}}{\sum_{j=1}^{M}{\left(\bar{f}\left(\eta_{j}\right)-\hat{f}\left(\eta_{j}\right)\right)}^{2}}, & \hat{f}\left(\eta_{j}\right)=\frac{1}{M}\sum_{j=1}^{M}f\left(\eta_{j}\right),\\ 1-\frac{\frac{1}{M}\sum_{j=1}^{M}{\left(\bar{\theta }\left(\eta_{j}\right)-\theta \left(\eta_{j}\right)\right)}^{2}}{\sum_{j=1}^{M}{\left(\bar{\theta }\left(\eta_{j}\right)-\hat{\theta }\left(\eta_{j}\right)\right)}^{2}}, & \hat{\theta }\left(\eta_{j}\right)=\frac{1}{M}\sum_{j=1}^{M}\theta \left(\eta_{j}\right),\\ 1-\frac{\frac{1}{M}\sum_{j=1}^{M}{\left(\bar{\phi }\left(\eta_{j}\right)-\phi \left(\eta_{j}\right)\right)}^{2}}{\sum_{j=1}^{M}{\left(\bar{\phi }\left(\eta_{j}\right)-\hat{\phi }\left(\eta_{j}\right)\right)}^{2}}, & \hat{\phi }\left(\eta_{j}\right)=\frac{1}{M}\sum_{j=1}^{M}\phi \left(\eta_{j}\right), \end{array}\right]}^{t}$$

(36) $$\left[ENSE_{f},ENSE_{\theta },ENSE_{\phi }\right]=\left[1-NSE_{f},1-NSE_{\theta },1-NSE_{\phi }\right].$$

Here, $\bar{f}$, $\bar{\theta }$, $\bar{\phi }$ and *f*, θ, ϕ are the analytical and approximate solutions, respectively. For the perfect modeling of solutions, the values of MAD and TIC approach zero.

**Figure 11:**
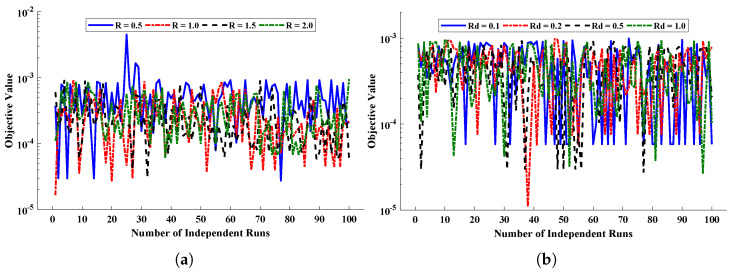
(**a**,**b**) The convergence of fitness value during the 100 independent runs of the designed technique.

**Figure 12:**
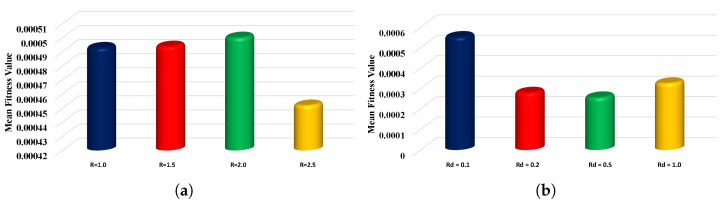
(**a**,**b**) The global values of fitness function during the 100 independent runs of the proposed ANN-AOA-IPA algorithm.

The behavior of the objective/fitness function given in Equation (23) is shown in Figure 11, and the global values of the fitness function are illustrated in Figure 12. In addition, a sensitivity analysis of the proposed algorithm was conducted by varying the population size or candidate space (Pop) and the number of neurons (k) in the ANN architecture. Table 4 shows that the accuracy of approximate solutions increases with the increase in the population size and number of neurons. The statistical results for the performance indicators in terms of minimum value, mean and standard deviation are reported in Table 5 and Table 6. The mean values of MAD, TIC and ENSE for each case are around $10^{-3}$ to $10^{-5}$, $10^{-4}$ to $10^{-5}$ and $10^{-4}$ to $10^{-7}$, respectively. The results demonstrate the accuracy of the results and the efficiency of the proposed algorithm in solving a mathematical model of the steady two-phase flow of a nanofluid in a semipermeable duct.

## 5. Conclusions

A mathematical model of the steady two-phase magnetohydrodynamic flow of a nanofluid in a semipermeable duct was analyzed. Moreover, to study the system, a metaheuristic-driven approach was designed based on the intelligent computational strength of artificial neural networks. ANNs were used to model the structure of approximate solutions for the velocity, temperature and concentration profiles of the nanofluid. Furthermore, unsupervised models of solutions known as objective functions were optimized with the hybridization of global and local search techniques. The designed ANN-AOA-IPA algorithm was successfully implemented to study the influence of variations in the thermophoretic parameter (Nt), Brownian (Nb) and radiation (Rd) motion parameters, Reynolds number (R), Eckert number (Ec), Hartmann number (Ha) and Schmidt number (Sc). The Nusselt number (Nu) and skin friction coefficient $C_{f}$ were calculated for different values of the Hartman number, Eckert number and suction parameter. The results illustrate that Nu and $C_{f}$ increase with the Hartman number and suction parameter, while an inverse relation is observed with the Eckert number. In addition, it is observed that the velocity, temperature and concentration profiles of the nanofluid increase with an increase in the suction parameter, Eckert number and Schmidt number, respectively. The results of ANN-AOA-IPA were compared with the Runge–Kutta method and machine learning algorithms, which reveal that the solutions obtained by the proposed technique overlap with the numerical solutions, with absolute errors of around $10^{-5}$ to $10^{-9}$. The stability, accuracy and efficiency of the designed technique were validated by error analysis based on MAD, TIC and ENSE. 

## Figures and Tables

**Table 1:** Appropriate parameter settings for the execution of AOA and interior point algorithm.

Algorithm	Parameters	Settings	Parameter	Settings
Arithmetic optimization algorithm	Lower bound	−5	Upper bound	5
	Search agents	80	Dimensions	90
	Maximum iterations	5000	Fitness	$\leq \;\;10^{-12}$
	Tolerance function	$\leq \;\;10^{-18}$	Tolerance constrained	$\leq \;\;10^{-18}$
Interior point algorithm	Lower bound	−5	Upper bound	5
	Maximum iterations	1000	Fitness	$\leq \;\;10^{-15}$
	Function evaluations	1,500,000	Tolerance function	$\leq \;\;10^{-20}$

**Table 2:** Comparison of approximate solutions for f(η) obtained by ANN-AOA-IPA with RK-4 method and stochastic machine learning algorithm.

η	Re=0.5				Re=1.5			
	Numerical	NN-BLM [45,46]	NARX-LM [47,48]	ANN-AOA-IPA	Numerical	NN-BLM [45,46]	NARX-LM [47,48]	ANN-AOA-IPA
−1	1.00000000	0.99996385	0.99999938	1.00000055	1.00000000	0.94071596	1.10009813	0.99999946
−0.9	1.07763894	1.08608331	1.07761050	1.07763906	1.07282631	1.07270067	1.10009797	1.07282754
−0.8	1.11587590	1.11489475	1.11586776	1.11587661	1.10009813	1.09571348	1.09998164	1.10009954
−0.7	1.12166425	1.11870199	1.12166471	1.12162656	1.09252600	1.08619716	1.09250160	1.09251966
−0.6	1.10084648	1.10025358	1.10034744	1.10084649	1.05832581	1.05931735	1.05836175	1.05837758
−0.5	1.05841043	1.06827881	1.05847989	1.05841021	1.00395629	1.00385653	1.00388772	1.00337256
−0.4	0.99868221	0.99875324	0.99874777	0.99868209	0.93461860	0.93460208	0.93467741	0.93460079
−0.3	0.92547167	0.92546679	0.92552296	0.92547108	0.85459585	0.99372664	0.85454526	0.85459395
−0.2	0.84218219	0.84218203	0.84220346	0.84218178	0.76748579	0.76748917	0.76746195	0.76748827
−0.1	0.75189435	0.75180907	0.75194013	0.75189332	0.67636211	0.67637960	0.67636574	0.67636559
0.0	0.65743016	0.65745095	0.65746523	0.65742961	0.58388820	0.58389632	0.58387412	0.58389402
0.1	0.56140356	0.32841222	0.56142094	0.56140284	0.49239957	0.49242261	0.49239969	0.49246100
0.2	0.46626097	0.46626563	0.46695412	0.46635119	0.40396540	0.40401405	0.40396405	0.40396402
0.3	0.37431537	0.37432027	0.37531147	0.37456661	0.32043680	0.32050855	0.32041108	0.32043193
0.4	0.28777627	0.28778534	0.28822966	0.28780743	0.24348682	0.24445067	0.24351486	0.24342219
0.5	0.20877774	0.20874843	0.20881059	0.20877765	0.17464595	0.13850070	0.17472544	0.17465424
0.6	0.13940632	0.13343492	0.13945447	0.13940621	0.11533566	0.11527305	0.11531411	0.11533162
0.7	0.08173037	0.08206249	0.08172059	0.08173037	0.06690252	0.05927328	0.06692670	0.06690254
0.8	0.03783243	0.03762772	0.03781267	0.03874270	0.03065445	0.03157715	0.03069898	0.03065476
0.9	0.00984625	0.01255599	0.01366272	0.00987969	0.00790112	0.06554558	0.00777912	0.00799663
1	0.00000000	0.00342423	0.00361723	0.00000143	0.00000006	0.03495787	0.00046412	−0.00002026

**Table 3:** Comparison of absolute error in the solutions obtained by ANN-AOA-IPA for different cases of steady phase flow of nanofluid.

η	Re=0.5			Re=1.5		
	NN-BLM	NARX-LM	ANN-AOA-IPA	NN-BLM	NARX-LM	ANN-AOA-IPA
−1	$3.6146\times 10^{-05}$	$6.1700\times 10^{-07}$	$5.4600\times 10^{-07}$	$5.9284\times 10^{-02}$	$1.0010\times 10^{-01}$	$5.3600\times 10^{-07}$
−0.9	$8.4444\times 10^{-03}$	$2.8437\times 10^{-05}$	$1.2400\times 10^{-07}$	$1.2564\times 10^{-04}$	$2.7272\times 10^{-02}$	$1.2270\times 10^{-06}$
−0.8	$9.8115\times 10^{-04}$	$8.1310\times 10^{-06}$	$7.1500\times 10^{-07}$	$4.3847\times 10^{-03}$	$1.1649\times 10^{-04}$	$1.4122\times 10^{-06}$
−0.7	$2.9623\times 10^{-03}$	$4.6400\times 10^{-07}$	$3.7684\times 10^{-05}$	$6.3288\times 10^{-03}$	$2.4406\times 10^{-05}$	$6.3390\times 10^{-06}$
−0.6	$5.9290\times 10^{-04}$	$4.9904\times 10^{-04}$	$1.2000\times 10^{-08}$	$9.9154\times 10^{-04}$	$3.5942\times 10^{-05}$	$5.1774\times 10^{-05}$
−0.5	$9.8684\times 10^{-03}$	$6.9465\times 10^{-05}$	$2.1700\times 10^{-07}$	$9.9768\times 10^{-05}$	$6.8573\times 10^{-05}$	$5.8373\times 10^{-04}$
−0.4	$7.1027\times 10^{-05}$	$6.5563\times 10^{-05}$	$1.1700\times 10^{-07}$	$1.6514\times 10^{-05}$	$5.8814\times 10^{-05}$	$1.7809\times 10^{-05}$
−0.3	$4.8780\times 10^{-06}$	$5.1293\times 10^{-05}$	$5.8100\times 10^{-07}$	$1.3913\times 10^{-01}$	$5.0592\times 10^{-05}$	$1.9030\times 10^{-06}$
−0.2	$1.5600\times 10^{-07}$	$2.1266\times 10^{-05}$	$4.1500\times 10^{-07}$	$3.3758\times 10^{-06}$	$2.3848\times 10^{-05}$	$2.4770\times 10^{-06}$
−0.1	$8.5271\times 10^{-05}$	$4.5785\times 10^{-05}$	$1.0200\times 10^{-06}$	$1.7495\times 10^{-05}$	$3.6397\times 10^{-06}$	$3.4880\times 10^{-06}$
0.0	$2.0789\times 10^{-05}$	$3.5065\times 10^{-05}$	$5.5000\times 10^{-07}$	$8.1227\times 10^{-06}$	$1.4083\times 10^{-05}$	$5.8220\times 10^{-06}$
0.1	$2.3299\times 10^{-01}$	$1.7381\times 10^{-05}$	$7.2200\times 10^{-07}$	$2.3036\times 10^{-05}$	$1.1355\times 10^{-07}$	$6.1425\times 10^{-05}$
0.2	$4.6580\times 10^{-06}$	$6.9315\times 10^{-04}$	$9.0217\times 10^{-05}$	$4.8650\times 10^{-05}$	$1.3485\times 10^{-06}$	$1.3820\times 10^{-06}$
0.3	$4.8940\times 10^{-06}$	$9.9610\times 10^{-04}$	$2.5124\times 10^{-04}$	$7.1757\times 10^{-05}$	$2.5717\times 10^{-05}$	$4.8660\times 10^{-06}$
0.4	$9.0670\times 10^{-06}$	$4.5339\times 10^{-04}$	$3.1165\times 10^{-05}$	$9.6385\times 10^{-04}$	$2.8036\times 10^{-05}$	$6.4633\times 10^{-05}$
0.5	$2.9312\times 10^{-05}$	$3.2851\times 10^{-05}$	$9.2000\times 10^{-08}$	$3.6145\times 10^{-02}$	$7.9499\times 10^{-05}$	$8.2900\times 10^{-06}$
0.6	$5.9714\times 10^{-03}$	$4.8149\times 10^{-05}$	$1.1500\times 10^{-07}$	$6.2606\times 10^{-05}$	$2.1551\times 10^{-05}$	$4.0370\times 10^{-06}$
0.7	$3.3212\times 10^{-04}$	$9.7760\times 10^{-06}$	$1.0000\times 10^{-09}$	$7.6292\times 10^{-03}$	$2.4182\times 10^{-05}$	$1.7000\times 10^{-08}$
0.8	$2.0470\times 10^{-04}$	$1.9754\times 10^{-05}$	$9.1027\times 10^{-04}$	$9.2270\times 10^{-04}$	$4.4529\times 10^{-05}$	$3.1400\times 10^{-07}$
0.9	$2.7097\times 10^{-03}$	$3.8165\times 10^{-03}$	$3.3443\times 10^{-05}$	$5.7644\times 10^{-02}$	$1.2200\times 10^{-04}$	$9.5513\times 10^{-05}$
1	$3.4242\times 10^{-03}$	$3.6172\times 10^{-03}$	$1.4281\times 10^{-06}$	$3.4958\times 10^{-02}$	$4.6406\times 10^{-04}$	$2.0318\times 10^{-05}$

**Table 4:** Sensitivity analysis of the proposed ANN-AOA-IPA algorithm by varying the size of the population space and increasing the number of neurons in ANN architecture.

η	Population Space			Number of Neurons		
	**Pop = 40**	**Pop = 60**	**Pop = 80**	**k = 10**	**k = 20**	**k = 30**
−1	$2.6864\times 10^{-03}$	$1.0329\times 10^{-04}$	$2.4641\times 10^{-06}$	$2.6253\times 10^{-03}$	$1.7346\times 10^{-04}$	$7.7574\times 10^{-07}$
−0.9	$5.8452\times 10^{-03}$	$3.0524\times 10^{-04}$	$4.6352\times 10^{-07}$	$7.1556\times 10^{-03}$	$5.5202\times 10^{-04}$	$3.0120\times 10^{-06}$
−0.8	$4.9577\times 10^{-04}$	$3.8561\times 10^{-06}$	$1.3737\times 10^{-06}$	$2.1806\times 10^{-03}$	$3.3220\times 10^{-04}$	$5.1192\times 10^{-06}$
−0.7	$3.9695\times 10^{-03}$	$1.8154\times 10^{-04}$	$1.9025\times 10^{-06}$	$5.4371\times 10^{-03}$	$2.2298\times 10^{-04}$	$2.8138\times 10^{-06}$
−0.6	$1.9468\times 10^{-03}$	$1.4502\times 10^{-03}$	$2.7830\times 10^{-05}$	$2.3706\times 10^{-03}$	$1.8001\times 10^{-04}$	$6.0552\times 10^{-05}$
−0.5	$1.7634\times 10^{-03}$	$8.2916\times 10^{-05}$	$1.7958\times 10^{-06}$	$2.0402\times 10^{-03}$	$2.7400\times 10^{-04}$	$5.2621\times 10^{-06}$
−0.4	$3.2420\times 10^{-03}$	$1.0875\times 10^{-04}$	$1.4003\times 10^{-07}$	$4.3142\times 10^{-03}$	$6.2096\times 10^{-05}$	$4.3763\times 10^{-06}$
−0.3	$1.5481\times 10^{-03}$	$1.4749\times 10^{-04}$	$1.2195\times 10^{-06}$	$3.6066\times 10^{-03}$	$1.6525\times 10^{-04}$	$9.4592\times 10^{-06}$
−0.2	$1.4065\times 10^{-03}$	$1.0883\times 10^{-03}$	$1.8041\times 10^{-09}$	$9.0876\times 10^{-04}$	$1.8397\times 10^{-04}$	$2.8093\times 10^{-06}$
−0.1	$2.9428\times 10^{-03}$	$2.8791\times 10^{-05}$	$1.5826\times 10^{-05}$	$2.0974\times 10^{-03}$	$7.0954\times 10^{-06}$	$8.1390\times 10^{-06}$
0	$1.8061\times 10^{-03}$	$4.8016\times 10^{-05}$	$8.2430\times 10^{-06}$	$3.9363\times 10^{-03}$	$2.2731\times 10^{-04}$	$1.0280\times 10^{-07}$
0.1	$9.8747\times 10^{-04}$	$8.8184\times 10^{-04}$	$7.0792\times 10^{-08}$	$3.8453\times 10^{-03}$	$2.6729\times 10^{-04}$	$3.5084\times 10^{-06}$
0.2	$3.0236\times 10^{-03}$	$7.9545\times 10^{-05}$	$7.2345\times 10^{-06}$	$1.9256\times 10^{-03}$	$4.9843\times 10^{-05}$	$1.1479\times 10^{-05}$
0.3	$2.3928\times 10^{-03}$	$3.1778\times 10^{-05}$	$8.9309\times 10^{-07}$	$9.7768\times 10^{-04}$	$2.9083\times 10^{-04}$	$9.8425\times 10^{-06}$
0.4	$6.1715\times 10^{-04}$	$2.9966\times 10^{-05}$	$5.5314\times 10^{-06}$	$3.5783\times 10^{-03}$	$4.6637\times 10^{-04}$	$5.0331\times 10^{-06}$
0.5	$3.2608\times 10^{-03}$	$7.4988\times 10^{-05}$	$8.4750\times 10^{-07}$	$4.5668\times 10^{-03}$	$2.3081\times 10^{-04}$	$1.4925\times 10^{-06}$
0.6	$2.2817\times 10^{-03}$	$7.7325\times 10^{-04}$	$6.1603\times 10^{-04}$	$3.1008\times 10^{-03}$	$3.5270\times 10^{-04}$	$3.1363\times 10^{-05}$
0.7	$2.2011\times 10^{-03}$	$2.5473\times 10^{-05}$	$5.9281\times 10^{-06}$	$6.4840\times 10^{-04}$	$7.4595\times 10^{-04}$	$1.8440\times 10^{-07}$
0.8	$3.7397\times 10^{-03}$	$6.3881\times 10^{-05}$	$1.6041\times 10^{-05}$	$4.8781\times 10^{-03}$	$1.8243\times 10^{-04}$	$1.2047\times 10^{-06}$
0.9	$4.1865\times 10^{-03}$	$1.1346\times 10^{-04}$	$9.6525\times 10^{-06}$	$5.4220\times 10^{-03}$	$1.1799\times 10^{-03}$	$2.9138\times 10^{-05}$
1	$1.3168\times 10^{-03}$	$9.7003\times 10^{-05}$	$7.0381\times 10^{-07}$	$5.1701\times 10^{-03}$	$4.8682\times 10^{-04}$	$1.1468\times 10^{-07}$

**Table 5:** Statistics of performance indicators in terms of minimum, mean and standard deviations obtained during 100 independent runs of the designed algorithm for the solution of f(η).

		Mean Absolute Deviations			Theil’s Inequality Coefficient			Error in Nash–Sutcliffe Efficiency		
		Minimum	Mean	Standard Deviation	Minimum	Mean	Standard Deviation	Minimum	Mean	Standard Deviation
f(η)	R=0.5	$7.2403\times 10^{-05}$	$3.5743\times 10^{-04}$	$2.9778\times 10^{-04}$	$4.6039\times 10^{-05}$	$5.1401\times 10^{-04}$	$3.5330\times 10^{-04}$	$7.3727\times 10^{-07}$	$2.2083\times 10^{-04}$	$2.6709\times 10^{-04}$
	R=1.0	$8.6929\times 10^{-05}$	$4.9791\times 10^{-04}$	$2.4997\times 10^{-04}$	$5.7010\times 10^{-05}$	$3.3104\times 10^{-04}$	$1.6783\times 10^{-04}$	$1.1169\times 10^{-06}$	$3.8924\times 10^{-05}$	$3.4451\times 10^{-05}$
	R=1.5	$1.9639\times 10^{-04}$	$4.0459\times 10^{-04}$	$2.3773\times 10^{-04}$	$1.3167\times 10^{-04}$	$2.7001\times 10^{-04}$	$1.5620\times 10^{-04}$	$6.0063\times 10^{-06}$	$3.3927\times 10^{-05}$	$3.7656\times 10^{-05}$
	R=2.0	$9.4110\times 10^{-04}$	$1.1000\times 10^{-03}$	$6.0482\times 10^{-05}$	$6.3155\times 10^{-04}$	$8.0922\times 10^{-04}$	$9.1869\times 10^{-05}$	$1.4547\times 10^{-04}$	$2.0401\times 10^{-04}$	$4.0899\times 10^{-05}$
θ(η)	R=0.5	$1.8540\times 10^{-04}$	$8.5374\times 10^{-04}$	$1.0000\times 10^{-03}$	$9.4233\times 10^{-05}$	$4.5948\times 10^{-04}$	$5.4992\times 10^{-04}$	$4.7044\times 10^{-06}$	$4.5903\times 10^{-04}$	$5.6429\times 10^{-04}$
	R=1.0	$1.1846\times 10^{-04}$	$5.2613\times 10^{-04}$	$2.1343\times 10^{-04}$	$9.5384\times 10^{-05}$	$4.4563\times 10^{-04}$	$2.0499\times 10^{-04}$	$3.6759\times 10^{-06}$	$1.0560\times 10^{-04}$	$9.7748\times 10^{-05}$
	R=1.5	$8.9358\times 10^{-05}$	$3.3748\times 10^{-04}$	$2.1856\times 10^{-04}$	$1.0725\times 10^{-04}$	$3.2449\times 10^{-04}$	$1.9673\times 10^{-04}$	$3.0354\times 10^{-06}$	$7.8839\times 10^{-05}$	$1.0081\times 10^{-04}$
	R=2.0	$1.5648\times 10^{-04}$	$4.1213\times 10^{-04}$	$1.7723\times 10^{-04}$	$1.8750\times 10^{-04}$	$4.8842\times 10^{-04}$	$2.0099\times 10^{-04}$	$1.1969\times 10^{-05}$	$9.7290\times 10^{-05}$	$6.5530\times 10^{-05}$
ϕ(η)	R=0.5	$2.6008\times 10^{-05}$	$2.9242\times 10^{-04}$	$1.8388\times 10^{-04}$	$2.6381\times 10^{-05}$	$2.6854\times 10^{-04}$	$1.6804\times 10^{-04}$	$1.8993\times 10^{-07}$	$2.6146\times 10^{-05}$	$2.4518\times 10^{-05}$
	R=1.0	$2.4168\times 10^{-05}$	$7.2382\times 10^{-05}$	$3.1487\times 10^{-05}$	$2.3046\times 10^{-05}$	$6.5648\times 10^{-05}$	$2.8007\times 10^{-05}$	$1.5679\times 10^{-07}$	$1.6672\times 10^{-06}$	$1.2839\times 10^{-06}$
	R=1.5	$3.4192\times 10^{-05}$	$5.6596\times 10^{-05}$	$1.6593\times 10^{-05}$	$3.2308\times 10^{-05}$	$5.0079\times 10^{-05}$	$1.4797\times 10^{-05}$	$3.0656\times 10^{-07}$	$9.0910\times 10^{-07}$	$5.1025\times 10^{-07}$
	R=2.0	$6.8627\times 10^{-05}$	$1.0628\times 10^{-04}$	$2.4705\times 10^{-05}$	$5.5882\times 10^{-05}$	$9.2091\times 10^{-05}$	$1.9497\times 10^{-05}$	$1.2165\times 10^{-06}$	$3.0670\times 10^{-06}$	$1.2993\times 10^{-06}$

**Table 6:** Statistics of performance indicators in terms of minimum, mean and standard deviations obtained during the 100 independent runs of the designed algorithm for the solution of θ(η).

		Mean Absolute Deviations			Theil’s Inequality Coefficient			Error in Nash–Sutcliffe Efficiency		
		Minimum	Mean	Standard Deviation	Minimum	Mean	Standard Deviation	Minimum	Mean	Standard Deviation
f(η)	Rd=0.1	$2.9129\times 10^{-04}$	$6.5088\times 10^{-04}$	$2.8805\times 10^{-04}$	$2.6609\times 10^{-04}$	$6.0289\times 10^{-04}$	$2.7352\times 10^{-04}$	$2.4264\times 10^{-05}$	$1.4013\times 10^{-04}$	$1.0901\times 10^{-04}$
	Rd=0.2	$5.2428\times 10^{-05}$	$1.3954\times 10^{-04}$	$6.5303\times 10^{-05}$	$4.6231\times 10^{-05}$	$1.3250\times 10^{-04}$	$6.7998\times 10^{-05}$	$7.8297\times 10^{-07}$	$6.5184\times 10^{-06}$	$5.1070\times 10^{-06}$
	Rd=0.5	$2.6008\times 10^{-05}$	$1.2272\times 10^{-04}$	$5.2551\times 10^{-05}$	$2.6381\times 10^{-05}$	$1.1161\times 10^{-04}$	$4.5722\times 10^{-05}$	$1.8993\times 10^{-07}$	$4.9182\times 10^{-06}$	$3.5645\times 10^{-06}$
	Rd=1.0	$4.9235\times 10^{-05}$	$1.7491\times 10^{-04}$	$1.3438\times 10^{-04}$	$4.2589\times 10^{-05}$	$1.9682\times 10^{-04}$	$1.6747\times 10^{-04}$	$6.7810\times 10^{-07}$	$1.0572\times 10^{-05}$	$1.6896\times 10^{-05}$
θ(η)	Rd=0.1	$6.0070\times 10^{-04}$	$9.1330\times 10^{-04}$	$2.4107\times 10^{-04}$	$8.2072\times 10^{-04}$	$1.0000\times 10^{-03}$	$1.5996\times 10^{-04}$	$1.0319\times 10^{-04}$	$3.1637\times 10^{-04}$	$1.3843\times 10^{-04}$
	Rd=0.2	$2.0447\times 10^{-04}$	$3.0581\times 10^{-04}$	$8.5580\times 10^{-05}$	$2.0198\times 10^{-04}$	$2.9700\times 10^{-04}$	$8.2541\times 10^{-05}$	$1.1910\times 10^{-05}$	$2.8308\times 10^{-05}$	$1.4805\times 10^{-05}$
	Rd=0.5	$1.2685\times 10^{-04}$	$2.3671\times 10^{-04}$	$9.9817\times 10^{-05}$	$1.1791\times 10^{-04}$	$2.1831\times 10^{-04}$	$9.1442\times 10^{-05}$	$4.5183\times 10^{-06}$	$1.8181\times 10^{-05}$	$1.4958\times 10^{-05}$
	Rd=1.0	$1.2235\times 10^{-04}$	$7.8569\times 10^{-04}$	$6.5424\times 10^{-04}$	$7.8651\times 10^{-04}$	$3.8227\times 10^{-04}$	$3.7916\times 10^{-04}$	$9.7866\times 10^{-06}$	$3.5243\times 10^{-05}$	$1.2779\times 10^{-05}$
ϕ(η)	Rd=0.1	$4.7175\times 10^{-04}$	$5.1762\times 10^{-04}$	$9.1730\times 10^{-05}$	$4.3889\times 10^{-04}$	$5.2589\times 10^{-04}$	$1.2690\times 10^{-04}$	$6.2487\times 10^{-05}$	$9.3344\times 10^{-05}$	$4.4357\times 10^{-05}$
	Rd=0.2	$5.5114\times 10^{-05}$	$7.2623\times 10^{-05}$	$2.6976\times 10^{-05}$	$4.0381\times 10^{-04}$	$4.6302\times 10^{-04}$	$7.5120\times 10^{-05}$	$4.4304\times 10^{-04}$	$5.0270\times 10^{-04}$	$8.6157\times 10^{-05}$
	Rd=0.5	$2.2756\times 10^{-05}$	$9.4426\times 10^{-05}$	$6.8102\times 10^{-05}$	$2.0798\times 10^{-05}$	$1.0284\times 10^{-04}$	$8.2923\times 10^{-05}$	$1.4485\times 10^{-07}$	$3.6473\times 10^{-06}$	$4.3006\times 10^{-06}$
	Rd=1.0	$3.7874\times 10^{-05}$	$3.5456\times 10^{-05}$	$1.8766\times 10^{-05}$	$9.6532\times 10^{-05}$	$9.8765\times 10^{-05}$	$1.5466\times 10^{-05}$	$6.4731\times 10^{-07}$	$9.5646\times 10^{-07}$	$4.5685\times 10^{-07}$

## Data Availability

The data that support the findings of this study are available from the corresponding author upon reasonable request.

## Nomenclature

AOA	Arithmetic optimization algorithm
IPA	Interior point algorithm
TIC	Theil’s inequality coefficient
MAD	Mean absolute deviation
NSE	Nash–Sutcliffe efficiency
ENSE	Error in Nash–Sutcliffe efficiency
Nt	Thermophoretic parameter
Ha	Hartmann number
Nb	Brownian motion parameter
Rd	Radiation motion parameter
Re	Reynolds number
Sc	Schmidt number
λ	Suction parameter
Ec	Eckert number
$C_{p}$	Specific heat capacity
Pr	Prandtl number
$B_{0}$	Magnetic field
$B_{R}$	Mean absorption coefficient
μ	Dynamic viscosity
Nu	Nusselt number
σ	Electrical conductivity
$C_{f}$	Skin friction coefficient
u,v	Horizontal and vertical velocities
η	Similarity-independent variable
$q_{r}$	Thermal radiation
θ	Dimensionless temperature
$\rho_{e}$	Stefan–Boltzmann constant
ϕ	Dimensionless concentration
*h*	Activation function
α,β,ω	Unknown neurons in ANN structure
*k*	Number of neurons
a,b,r1,r2,r3	Random numbers
$UB_{j},LB_{j}$	Upper and lower bounds
$\bar{\mu }$	Controlling parameter in AOA
$\bar{\alpha }$	Sensitivity parameter in AOA
f, p (subscripts)	Fluid phase, particulate phase

## Appendix A

Approximate series solutions for the velocity, temperature and concentration of the fluid for R=0.5 are given as

(A1) $$\begin{array}{cc} f(\eta )=\; & \frac{4.61369980524645}{1+e^{-(5.08711787637231\eta +9.90867776328236)}}\\ \; & +\frac{2.93758362572738}{1+e^{-(3.09195460470904\eta +5.69916567641777)}}\\ \; & +\frac{3.29945980406045}{1+e^{-(2.32570116204802\eta +4.02631566205142)}}\\ \; & +\frac{-3.78192366991081}{1+e^{\left(1.62985653339985\eta -5.49858809617563\right)}}\\ \; & +\frac{-3.22583732511908}{1+e^{-(-0.370478023140672\eta +2.00839985002585)}}\\ \; & +\frac{-5.34813467606133}{1+e^{-(-2.92997660113540\eta +6.77439711326103)}}\\ \; & +\frac{-1.74596240330998}{1+e^{-(1.62626182324585\eta +0.401259099155673)}}\\ \; & +\frac{0.0285998274897956}{1+e^{-(2.71661052797685\eta +0.432623409646409)}}\\ \; & +\frac{-7.04187260919816}{1+e^{-(1.09619593671067\eta -1.45607700580778)}}\\ \; & +\frac{6.32391288523611}{1+e^{-(1.50299659396555\eta -2.60292294090048)}}, \end{array}$$

(A2) $$\begin{array}{cc} \theta (\eta )=\; & \frac{-0.762329897926404}{1+e^{-(1.68494525972436\eta +31.2426609585497)}}\\ \; & +\frac{-2.31189639017061}{1+e^{-(-1.50469611836172\eta +1.56501407903514)}}\\ \; & +\frac{-2.66393004854463}{1+e^{-(-1.06620117001768\eta -1.80630124000031)}}\\ \; & +\frac{-6.14157555274085}{1+e^{-(2.16908279747484\eta -1.09700349256711)}}\\ \; & +\frac{5.47586997864664}{1+e^{-(-5.59059045698216\eta -7.19784783962392)}}\\ \; & +\frac{-6.82815233359154}{1+e^{-(1.34558295675714\eta -3.70915380122379)}}\\ \; & +\frac{-3.11479387823252}{1+e^{-(-0.0884720225553450\eta -1.06256782303172)}}\\ \; & +\frac{8.60883142974415}{1+e^{-(5.61401069041918\eta +8.01296209229107)}}\\ \; & +\frac{-3.00270903621335}{1+e^{-(-2.49238263574495\eta +1.11606951601859)}}\\ \; & +\frac{0.914452301076381}{1+e^{-(-1.58802329749731\eta -4.04995378577328)}}, \end{array}$$

(A3) $$\begin{array}{cc} \Phi (\eta )=\; & \frac{-4.29104167184275}{1+e^{-(1.29160502365402\eta +0.936652114155683)}}\\ \; & +\frac{-4.95499508899306}{1+e^{-(-0.619641753235744\eta +2.37818424387284)}}\\ \; & +\frac{7.50577678885757}{1+e^{-(0.607093211631663\eta +1.19078083137004)}}\\ \; & +\frac{-5.32284504861338}{1+e^{-(0.539662722456454\eta +1.56094286041994)}}\\ \; & +\frac{3.55841274393489}{1+e^{-(0.414796532515809\eta -0.316209500021666)}}\\ \; & +\frac{-4.70911002560639}{1+e^{-(1.67967325760117\eta -2.99941621832991)}}\\ \; & +\frac{-5.20837123119641}{1+e^{-(-1.23434514770494\eta +3.11287353056052)}}\\ \; & +\frac{10.0237940205905}{1+e^{-(-0.332933782284494\eta -2.03015694260553)}}\\ \; & +\frac{9.18640377585145}{1+e^{-(3.27340937126084\eta +5.53181041917603)}}\\ \; & +\frac{7.84723429468485}{1+e^{-(-3.67127578557170\eta -5.92860850158342)}}. \end{array}$$
